# Development of a novel monoclonal antibody with reactivity to a wide range of Venezuelan equine encephalitis virus strains

**DOI:** 10.1186/1743-422X-6-206

**Published:** 2009-11-19

**Authors:** Lyn M O'Brien, Cindy D Underwood-Fowler, Sarah A Goodchild, Amanda L Phelps, Robert J Phillpotts

**Affiliations:** 1 Biomedical Sciences Department, Defence Science and Technology Laboratory, Porton Down, Salisbury, Wiltshire, SP4 0JQ, UK

## Abstract

**Background:**

There is currently a requirement for antiviral therapies capable of protecting against infection with Venezuelan equine encephalitis virus (VEEV), as a licensed vaccine is not available for general human use. Monoclonal antibodies are increasingly being developed as therapeutics and are potential treatments for VEEV as they have been shown to be protective in the mouse model of disease. However, to be truly effective, the antibody should recognise multiple strains of VEEV and broadly reactive monoclonal antibodies are rarely and only coincidentally isolated using classical hybridoma technology.

**Results:**

In this work, methods were developed to reliably derive broadly reactive murine antibodies. A phage library was created that expressed single chain variable fragments (scFv) isolated from mice immunised with multiple strains of VEEV. A broadly reactive scFv was identified and incorporated into a murine IgG2a framework. This novel antibody retained the broad reactivity exhibited by the scFv but did not possess virus neutralising activity. However, the antibody was still able to protect mice against VEEV disease induced by strain TrD when administered 24 h prior to challenge.

**Conclusion:**

A monoclonal antibody possessing reactivity to a wide range of VEEV strains may be of benefit as a generic antiviral therapy. However, humanisation of the murine antibody will be required before it can be tested in humans.

Crown Copyright © 2009

## Background

The *Alphavirus *Venezuelan equine encephalitis virus (VEEV) is a single stranded, positive-sense RNA virus maintained in nature in a cycle between small rodents and mosquitoes [1]. Six serogroups (I-VI) are currently recognised within the VEEV complex. Spread of epizootic strains of the virus (IA/B and IC) to equines leads to a high viraemia followed by lethal encephalitis and lateral spread to humans. In the human host, VEEV can produce a febrile illness followed in a small proportion of cases by severe encephalitis. Equine epizootics may lead to widespread outbreaks of human encephalitis involving thousands of cases and hundreds of deaths [1]. Viruses in other serogroups do not appear to be equine-virulent and persist in a stable enzootic cycle. Natural transmission of enzootic viruses to humans is rare but may be associated with severe disease [2].

Epizootic VEEV can be controlled by the immunisation of equines with the attenuated vaccine strain TC-83. Although TC-83 is solidly protective in equines and has a good safety record [2], in humans it fails to produce protective immunity in up to 20% of recipients and is reactogenic in around 20% of recipients [3]. There have also been reports that the vaccine is potentially diabetogenic [4] and teratogenic [5]. Consequently, TC-83 is no longer available for human use in Europe and has limited availability in the U.S.A [6]. Both epizootic and enzootic strains of VEEV are infectious for humans by the airborne route and have been responsible for a number of laboratory infections [7].

In the absence of a suitable vaccine, antiviral therapies which are effective in prophylaxis and treatment of VEEV infection are required. There is evidence to suggest that protection against VEEV requires high antibody levels and, in the case of airborne infection, the presence of antibody on the mucosal surface of the respiratory tract [8]. Previous studies in the mouse model have shown that monoclonal antibodies can protect against VEEV and are effective against disease even when administered 24 h after exposure [8-10]. Although broadly reactive murine monoclonal antibodies have been coincidentally isolated using classical hybridoma technology [10], in general monoclonal antibodies have narrow specificities which limit their use as antiviral therapies. We set out to develop a capability to reliably derive new broadly reactive antibodies in the mouse, which would have the potential to protect humans against exposure to a range of VEEV strains.

## Results

### Generation of a novel VEEV-specific monoclonal antibody

**Figure 1:**
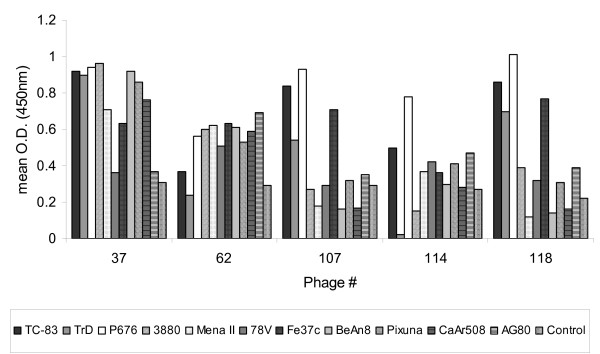
**Reactivity of phagemid clones to a wide range of VEEV strains**. Supernatants from phagemid clones, containing equivalent bacteriophage titres, were tested by ELISA using antigen prepared from VEEV strains TC-83, TrD, P676, 3880, Mena II, 78V, Fe37c, BeAn8, Pixuna, CaAr508 and AG80 (subtypes IA/B, IA/B, IC, ID, IE, IF, II, IIIA, IV, V and VI respectively). Negative control antigen was prepared from cells that had been mock infected. n = 3 for all data points.

**Figure 2:**
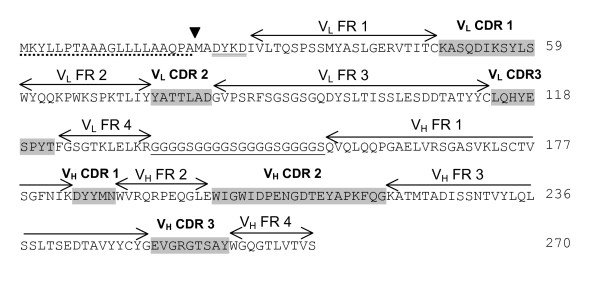
**Annotated amino acid sequence of scFv CUF37-2a**. Nucleotide sequences were edited and translated using Lasergene software http://www.dnastar.com. The Pel B leader peptide to direct secretion of the scFv to the periplasm of *E. coli *host cells during heterologous expression is underscored with a dotted line. The cleavage point of this signal peptide is indicated with a block arrow. The presence of a Flag-tag antibody at the N-terminus of the protein is shown with a double underline. The poly-glycine linker joining the V_L _and V_H _chains of the scFv is underlined with a single solid line. The Framework Regions (FR) and Complementarity Determining Regions (CDR) within the scFv sequence are indicated with arrows and with shading respectively.

Balb/c mice were initially immunised with VEEV vaccine strain TC-83, which is known to provide solid protection against a large challenge dose of most, if not all, mouse-virulent VEEV strains. Two doses of a mixture of representative viruses from subtypes IA/B, IC, ID, IE, IF, II, IIIA, IV, V and VI were then administered to the immune mice on days 14 and 21. The anti-VEEV immune response was assessed on day 28 (end-point titre greater than 1:500 000) and the spleens removed for extraction of RNA and conversion to cDNA. This was used to create a phage library expressing single chain variable fragments (scFv) which was enriched for antigen-specific scFv by two rounds of panning with antigen from VEEV strain TC-83. Individual phagemid clones were then tested for reactivity to strain TC-83 by ELISA and positive clones were assessed for uniqueness by analysing restriction digest patterns. Eight unique clones were sequenced and compared at the amino acid level for homology. A low level of homology was found between the scFv sequences indicating that the response to VEEV is not oligoclonal. Six of the unique clones were tested by ELISA for reactivity to multiple VEEV strains (Figure 1). Phagemid clone #12 is not shown in Figure 1 as it had a high level of reactivity to the negative control antigen and therefore conclusions can not be made with regard to VEEV reactivity. Phagemid clone #37 showed the highest level of activity to the widest range of strains and a low reactivity to the negative control antigen. It was therefore chosen for conversion into a murine IgG2a kappa antibody, which was designated CUF37-2a. Murine IgG2a was chosen as the framework as it has equivalent biological and functional activities to human IgG1. The amino acid sequence of the scFv incorporated into CUF37-2a is shown in Figure 2.

### Activity of CUF37-2a in ELISA and Neutralisation assays

**Figure 3:**
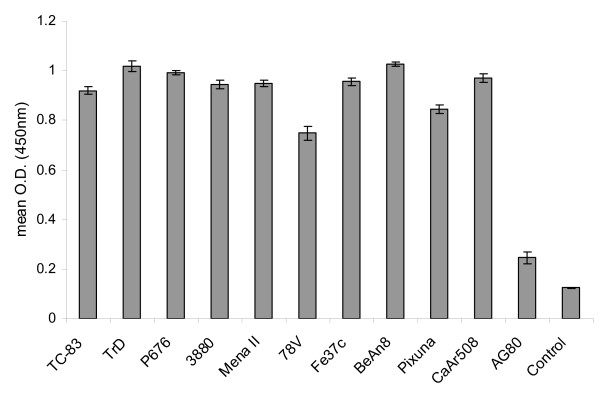
**Reactivity of CUF37-2a to multiple VEEV strains**. CUF37-2a (20 μg/ml) was tested by ELISA using antigen prepared from VEEV strains TC-83, TrD, P676, 3880, Mena II, 78V, Fe37c, BeAn8, Pixuna, CaAr508 and AG80 (subtypes IA/B, IA/B, IC, ID, IE, IF, II, IIIA, IV, V and VI respectively). Negative control antigen was prepared from cells that had been mock infected. n = 6 for all data points, 95% confidence intervals are shown.

In order to ensure that the range of VEEV reactivity had been retained during the incorporation of scFv from phagemid clone #37 into CUF37-2a, the antibody was tested in an ELISA using antigens from multiple strains (Figure 3). High levels of reactivity were seen for all strains, with the exception of AG80 (subtype VI) but phagemid clone #37 did not react well with this strain either (Figure 1). However, when the ability of the antibody to neutralise virus infectivity was tested, it was found that CUF37-2a was not able to neutralise virus from subtypes IA/B (strain TrD), II (strain Fe37c) or III (strain BeAn8) (results not shown).

### Glycoprotein specificity

**Figure 4:**
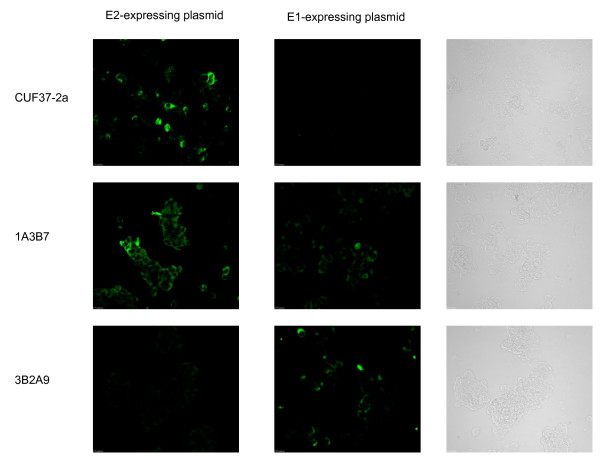
**CUF37-2a reacts with the VEEV E2 glycoprotein**. HEK 293 cells were transfected with plasmids expressing either the E2 or E1 glycoprotein of VEEV. 48 h later, the cells were fixed and reacted with 10 μg/ml CUF37-2a, 1A3B7 (E2-specific) or 3B2A9 (E1-specific) followed by anti-mouse IgG-FITC. The first two columns show representative fields of view under UV illumination. The third column shows identical brightfield views of negative UV-illuminated views.

VEEV has two major glycoproteins (E2 and E1) that occur on the virus surface as a heterodimer. Antibody reactivity to either protein may be associated with protection against virus challenge. When tested, CUF37-2a reacted with cells expressing the E2 glycoprotein but not with cells expressing the E1 glycoprotein (Figure 4), indicating that the antibody is specific for the viral E2 protein rather than the E1 protein. As expected, the E2-specific antibody (1A3B7) and E1-specific antibody (3B2A9) reacted with cells expressing the appropriate glycoprotein (Figure 4).

### Passive protection

Previous work has demonstrated that monoclonal antibodies which possess virus neutralising activity are effective at protecting mice from VEEV challenge [10]. However, protection in vivo is not necessarily associated with the ability of antibodies to neutralise virus. Functions of the Fc region of the antibody also play a role, principally the capacity to bind to macrophage Fc receptors [10,11]. It was therefore decided to test the ability of CUF37-2a to protect mice against VEEV strain TrD (subtype IA/B).

**Figure 5:**
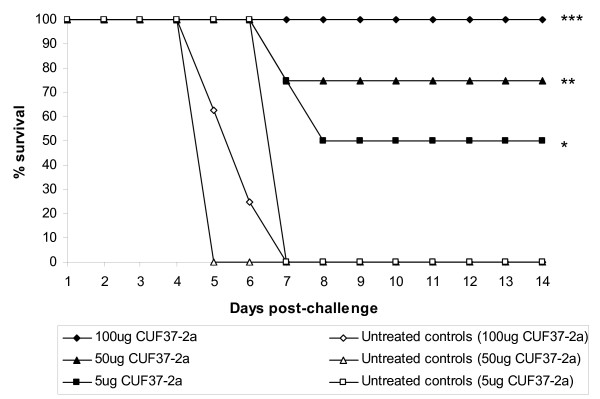
**CUF37-2a protects against VEEV disease when administered 24h prior to challenge**. In three independent experiments, Balb/c mice (7-8mice/group) remained untreated or were injected with CUF37-2a (5, 50 or 100 μg) intraperitoneally. 100LD_50 _VEEV strain TrD were administered subcutaneously 24h later. After challenge, mice were observed twice daily for clinical signs of infection and were culled when appropriate using humane endpoints. *P = 0.0043, **P = 0.0001 and ***P < 0.0001, Mantel-Maenszel Logrank test.

In three independent experiments (using the same stock of virus for challenge), the ability of a range of doses of CUF37-2a to protect against VEEV disease was assessed (Figure 5). Untreated mice did not survive the challenge dose and the median time to death was six days. The enhanced survival observed when mice were treated with CUF37-2a was statistically significant compared to untreated mice (P = 0.0043, P = 0.0001 and P < 0.0001 with 5, 50 and 100 μg CUF37-2a respectively). The increases in survival rates observed when a larger dose of antibody was administered to mice was not significant (P = 0.1139), although surviving mice treated with 50 or 100 μg CUF37-2a showed no clinical signs of infection whereas all mice treated with 5 μg CUF37-2a exhibited some clinical signs. However, it was determined by regression analysis that the relationship between survival and antibody concentration was significant (P = 0.0371). From the regression equation, 50% protection was achieved with a dose of 9.15 μg CUF37-2a.

The sera of mice that had been treated with 100 μg CUF37-2a were tested for VEEV-specific IgG1 by ELISA. The levels of IgG1 were measured in order to distinguish the response induced by the murine immune system and CUF37-2a, which is IgG2a. All mice generated an immune response to VEEV (mean 232.39 ng/ml, 95% confidence interval 106.94 ng/ml, n = 8). However, it is not known if this response had a role to play in the survival of mice treated with CUF37-2a. The brains of mice that had been treated with 100 μg CUF37-2a were also harvested and tested for the presence of virus. No virus was detectable in any of the brains (n = 8) whereas brains that were harvested from untreated mice culled 7 days after challenge (n = 2) contained 2.967 × 10^7^pfu and 5.368 × 10^7^pfu.

## Discussion

Effective antiviral therapies are required for VEEV as a vaccine is not generally available. Monoclonal antibodies are finding increasing application for therapies against other viruses [12] and they have been shown to be protective in the mouse model of VEEV disease [8-10]. This is the first demonstration of a monoclonal antibody, specifically designed to be reactive against multiple VEEV strains, being created using phage display technology and molecular biology techniques.

The purpose of this work was to create a novel antibody able to react with a wide range of VEEV strains which would have potential as an antiviral therapy for human use. Murine antibodies generally require molecular manipulation to make them similar to human antibodies (a process known as humanisation) before they can be used as therapeutics in humans. Human VEEV-specific monoclonal antibodies, produced by phage display technology [13], have not yet proved to be broadly cross-reactive and the hyperimmunisation regimes necessary to ensure a high frequency of broadly reactive antibodies would not be ethical in humans. We therefore believe that developing broadly reactive antibodies in mice and then subjecting the antibodies to humanisation procedures is more likely to lead to the development of an anti-VEEV therapy suitable for humans.

For therapeutic applications, antibodies with virus neutralising activity have usually been selected. As CUF37-2a was not able to neutralise the infectivity of multiple subtypes (IA/B, II or III), the antibody was only tested against VEEV strain TrD. A single dose successfully protected mice when administered 24 h prior to challenge. The protective activity of CUF37-2a may have been due to the ability of the antibody to abort the infection, to prevent spread of virus to the brain or to delay virus replication, giving the host immune response time to respond and control virus infection. It was determined that 50% of mice would be protected from a subcutaneous VEEV challenge when a dose of 9.15 μg of CUF37-2a was administered 24 h prior to challenge. Previous work [8,10] has shown that other VEEV-specific monoclonal antibodies (1A4A-1, 3B2A-9 and 1A3B-7) protect 50% of Balb/c mice against an airborne challenge at doses of 8, 10 and 10 μg respectively. Although CUF37-2a was not neutralising, protection induced by this antibody, which was generated using a phage library and molecular incorporation into an IgG2a framework, seems to compare favourably to protection induced by antibodies generated using classical hybridoma technology (1A4A-1, 3B2A-9 and 1A3B-7). Humanisation of CUF37-2a will be essential if this antibody is to find use as an antiviral in humans and these data suggest that CUF37-2a may be a suitable candidate.

The pathogenesis of VEEV disease in mice and humans is believed to be similar and in mice the virus usually enters the central nervous system two or three days after peripheral inoculation [14]. After airborne infection there is the additional possibility that virus may multiply in the olfactory neuroepithelium and thereby gain direct access to the olfactory nerve and brain. Thus, there is limited time available for antivirals to be administered after exposure to VEEV if they are to be used as therapeutics rather than as prophylactics. In previous work, monoclonal antibodies used as post-exposure antiviral therapies for VEEV were only effective when administered 24 h after infection [8,9] and not at 48 h [9] or 72 h [8]. Antivirals therefore need to be administered quickly enough after infection to prevent VEEV from accessing the brain or, alternatively, antivirals that are able to cross the blood-brain barrier are required to block viral replication in the brain. Previously, intraperitoneally administered monoclonal antibodies have been shown to have little effect on established VEEV infection of the brain [8] indicating that specialised delivery systems will be necessary to transport them into the brain in order to inhibit established infections and prevent encephalitis.

## Conclusion

In the present study, we have developed methods that use phage display technology in order to generate a monoclonal antibody with activity against a wide range of VEEV strains. The ability to reliably derive broadly reactive antibodies in the mouse is a significant improvement on depending on their chance isolation when classical hybridoma technology is used. Monoclonal antibodies are attractive candidates for new antiviral therapies for VEEV and an antibody capable of reacting with multiple strains would be the most desirable. However, before administration to humans, it is likely that an antibody generated in the mouse would have to undergo a degree of humanisation so that adverse immune reactions are avoided.

## Materials and methods

### Cells and viruses

The L929 (murine fibroblast), HEK 293 (human kidney) and Vero (simian kidney) cell lines (European Collection of Animal Cell Cultures, U.K.) were propagated by standard methods using the recommended culture media. Stocks of VEEV vaccine strain TC-83 were propagated from a vial of vaccine originally prepared for human use (National Drug Company, Philadelphia, U.S.A.). Strains of VEEV from serogroups IA/B (Trinidad donkey; TrD), IC (P676), ID (3880), IE (Mena II), IF (78V), II (Fe37c), IIIA (BeAn8), IV (Pixuna), V (CaAr508) and VI (AG80) were kindly supplied by Dr. R.E. Shope (University of Texas Medical Branch, U.S.A.). Virulent virus stocks were prepared and the titre determined as described by Phillpotts [10]. All work with virulent VEEV was carried out under U.K. Advisory Committee on Dangerous Pathogens Level 3 containment.

### Generation of a VEEV-reactive scFv phage library and conversion of one clone into a monoclonal antibody

Balb/c mice (7-9 weeks old, Charles River, U.K.) were immunised subcutaneously with 10^5 ^pfu of vaccine strain TC-83. On days 14 and 21, mice were immunised subcutaneously with a mixture of VEEV strains (TrD, P676, 3880, Mena II, 78V, Fe37c, BeAn8, Pixuna, CaAr508 and AG80; subtypes IA/B, IC, ID, IE, IF, II, IIIA, IV, V and VI respectively), totalling approximately 10^6^LD_50 _(approximately 10^6 ^pfu). Serum samples were taken from the marginal tail vein on day 28 and assayed for an anti-VEEV polyclonal response by ELISA with β propiolactone-inactivated TC-83 antigen [15]. Spleens from five immune mice were removed and processed to extract RNA (TRIzol^® ^Reagent, Invitrogen, U.K.) which was then converted to cDNA (SuperScript^® ^III Reverse Transcriptase, Invitrogen). Antibody heavy- and light-chain-specific primers were used in a PCR reaction to generate pools of heavy- and light-chain DNA from the cDNA template [16]. Single chain V_L_-Linker-V_H _constructs (scFv) were then produced using overlap extension PCR with specific single chain primers incorporating a linker region [16]. Purified single chain DNA was digested (Sfi I; New England Biolabs, U.S.A.) and ligated into pAK100 vector [17]. Phagemids were electroporated into *E.coli *XL1-Blue (Stratagene, U.S.A.) to produce a library of unique clones. Specificity of the library was increased by two rounds of biopanning [18] against β propiolactone-inactivated antigen from strain TC-83. Single colonies were isolated from stock obtained from the second round of panning and phage supernatants produced from these clones were assayed by ELISA with β propiolactone-inactivated TC-83 antigen and HRP-conjugated mouse anti-phage M13 (Amersham Pharmacia Biotech, U.K.) as the secondary antibody. Absorbance values greater than twice the background level were deemed to be positive. The scFv gene fragments from the positive phagemid clones were amplified using PCR and initially assessed for uniqueness by analysing restriction digest patterns (BstN I; New England Biolabs, U.S.A.). Those clones regarded as unique were analysed by DNA sequencing and compared at the amino acid level for homology. The supernatants from six phagemid clones, containing equivalent bacteriophage titres, were chosen for analysis by ELISA using sucrose density gradient-purified antigen from multiple VEEV strains (TrD, P676, 3880, Mena II, 78V, Fe37c, BeAn8, Pixuna, CaAr508 and AG80) and HRP-conjugated mouse anti-phage M13 as the secondary antibody. The scFv from the clone exhibiting the strongest, most wide-ranging response was converted into a full murine IgG2a kappa antibody (Haptogen, U.K.). This novel antibody was designated CUF37-2a and a purified stock of the antibody was supplied by Haptogen.

### Testing the activity of CUF37-2a in vitro

The ability of CUF37-2a (20 μg/ml) to recognise a variety of VEEV strains was tested by ELISA using sucrose density gradient-purified antigen from strains TrD, P676, 3880, Mena II, 78V, Fe37c, BeAn8, Pixuna, CaAr508 and AG80. So that the reactivity could be meaningfully compared, the VEEV antigens used in the ELISA were first examined by SDS-PAGE and scanning densitometry. Each antigen was diluted in coating buffer to contain an equivalent amount of virus glycoprotein. The ability of the antibody to neutralise virus infectivity was also determined. CUF37-2a (25 μg) was mixed with VEEV strains TrD, Fe37c or BeAn8 (approximately 100 pfu) and incubated at 4°C overnight. Residual infectious virus was estimated by plaque assay in L929 cells. A reduction in plaque numbers (compared to virus control wells) of equal to or greater than 50% in wells inoculated with the virus plus antibody mixture was considered indicative of neutralisation.

### Assessing the glycoprotein specificity of CUF37-2a

The capacity of CUF37-2a to bind to the VEEV E2 or E1 glycoprotein was determined by immunofluorescence staining. Plasmids expressing either the E2 or E1 protein from strain TrD (GenBank accession number J04332) were constructed by GeneArt (Germany). HEK 293 cells were transfected with each plasmid using the transfection reagent Lipofectamine 2000 (Invitrogen, UK), according to the manufacturer's guidelines. The cells were fixed in acetone after 48 h and were incubated with 10 μg/ml CUF37-2a, 1A3B7 (E2-specific monoclonal antibody, a kind gift of Dr. J.T. Roehrig, Division of Vector-borne Infectious Diseases, CDC, Fort Collins, Colorado, U.S.A.; Phillpotts, 2006) or 3B2A9 (E1-specific monoclonal antibody, a kind gift of Dr. J.T. Roehrig; Phillpotts, 2006) followed by a 1/800 dilution of anti-mouse IgG conjugated to FITC (Sigma, U.K.) before being examined under UV illumination.

### Determining the in vivo activity of CUF37-2a

The ability of CUF37-2a to protect against a challenge dose of 100LD_50 _(approximately 30-50 pfu) VEEV strain TrD (subtype IA/B) was tested. In three independent experiments, groups of Balb/c mice (7-9 weeks old, Charles River, U.K.) remained untreated or were injected intraperitoneally with 5, 50 or 100 μg of antibody in 50-100 μl PBS. The challenge virus was administered subcutaneously 24 h later. After challenge, mice were observed twice daily for clinical signs of infection by an independent observer [15]. Humane endpoints were used and these experiments therefore record the occurrence of severe disease rather than mortality [19]. Even though it is rare for animals infected with virulent VEEV and showing signs of severe illness to survive, our use of humane endpoints should be considered when interpreting any virus dose expressed here as 50% lethal doses (LD_50_).

### Enzyme immunoassay

Mouse sera, harvested by cardiac puncture 14 days after the challenge dose was administered, were assayed for VEEV-specific IgG1 antibodies using sucrose density gradient-purified antigen from strain TrD [10]. Immunoglobulin concentrations were estimated by comparison of the absorbance values generated by diluted serum samples (three replicates) with a standard curve prepared from dilutions of mouse IgG1 (Sigma, U.K.).

### Titration of virus in the brain

The amount of VEEV strain TrD present within mouse brains was determined by titration on Vero cells. Brains were removed and homogenised in 2 ml PBS by passing through a 70 μm nylon cell strainer (BD Falcon, U.K.). 200 μl of the cell suspension were added to each well of the first column of a 96-well plate and the homogenate was then serially diluted (1:10) in cell culture media across the plate. 100 μl of the diluted homogenate from each well were then added to the corresponding well of a 96-well plate containing confluent monolayers of Vero cells. The cells were incubated for 72 h after which time the monolayers were fixed by the addition of 10% (v/v) formal saline and stained with 0.1% (w/v) crystal violet. The concentration of VEEV, expressed as 50% tissue culture infectious doses (TCID_50_), was calculated by Reed-Muench analysis of virus-positive wells [20]. The concentration was then converted to pfu by multiplying the TCID_50 _value by 0.69 [21].

### Statistical methods

Statistical analysis was performed using the Mantel-Maenszel Logrank test and GraphPad Prism http://www.graphpad.com software.

## Competing interests

The authors declare that they have no competing interests.

## Authors' contributions

CDU-F, SAG and RJP generated the scFv phage library and tested it in vitro. LMOB tested antibody activity in vitro and determined the specificity. ALP carried out the animal study and LMOB tested the samples harvested from mice. RJP conceived of the study and LMOB drafted the manuscript. All authors read, contributed to and approved the final manuscript.
